# Selection and Validation of Appropriate Reference Genes for Quantitative Real-Time PCR Analysis of Gene Expression in *Lycoris aurea*

**DOI:** 10.3389/fpls.2016.00536

**Published:** 2016-04-25

**Authors:** Rui Ma, Sheng Xu, Yucheng Zhao, Bing Xia, Ren Wang

**Affiliations:** ^1^Institute of Botany, Jiangsu Province and Chinese Academy of SciencesNanjing, China; ^2^State Key Laboratory of Natural Medicines, Department of Natural Medicinal Chemistry, China Pharmaceutical UniversityNanjing, China

**Keywords:** gene quantification, quantitative real-time PCR (qRT-PCR), reference gene, abiotic stress, *Lycoris aurea*

## Abstract

*Lycoris aurea* (L' Hér.) Herb, a perennial grass species, produces a unique variety of pharmacologically active Amaryllidaceae alkaloids. However, the key enzymes and their expression pattern involved in the biosynthesis of Amaryllidaceae alkaloids (especially for galanthamine) are far from being fully understood. Quantitative real-time polymerase chain reaction (qRT-PCR), a commonly used method for quantifying gene expression, requires stable reference genes to normalize its data. In this study, to choose the appropriate reference genes under different experimental conditions, 14 genes including *YLS8* (*mitosis protein YLS8*), *CYP2* (*Cyclophilin 2*), *CYP 1* (*Cyclophilin 1*), *TIP41* (*TIP41-like protein*), *EXP2* (*Expressed protein 2*), *PTBP1* (*Polypyrimidine tract-binding protein 1*), *EXP1* (*Expressed protein 1*), *PP2A* (*Serine/threonine-protein phosphatase 2A*), β*-TUB* (β*-tubulin*), α*-TUB* (α*-tubulin*), *EF1-*α (*Elongation factor 1-*α), *UBC* (*Ubiquitin-conjugating enzyme*), *ACT* (*Actin*) and *GAPDH* (*Glyceraldehyde 3-phosphate dehydrogenase*) were selected from the transcriptome datasets of *L. aurea*. And then, expressions of these genes were assessed by qRT-PCR in various tissues and the roots under different treatments. The expression stability of the 14 candidates was analyzed by three commonly used software programs (geNorm, NormFinder, and BestKeeper), and their results were further integrated into a comprehensive ranking based on the geometric mean. The results show the relatively stable genes for each subset as follows: (1) *EXP1* and *TIP41* for all samples; (2) *UBC* and *EXP1* for NaCl stress; (3) *PTBP1* and *EXP1* for heat stress, polyethylene glycol (PEG) stress and ABA treatment; (4) *UBC* and *CYP2* for cold stress; (5) *PTBP1* and *PP2A* for sodium nitroprusside (SNP) treatment; (6) *CYP1* and *TIP41* for methyl jasmonate (MeJA) treatment; and (7) *EXP1* and *TIP41* for various tissues. The reliability of these results was further enhanced through comparison between part qRT-PCR result and RNA sequencing (RNA-seq) data. In summary, our results identified appropriate reference genes for qRT-PCR in *L. aurea*, and will facilitate gene expression studies under these conditions.

## Introduction

*Lycoris aurea* (L' Hér.) Herb, also called Golden Magic Lily, is an ornamentally and medicinally important species of the Amaryllidaceae family. It belonged to the genus *Lycoris* which composed of approximately 20 species of flowering plants native to the moist warm temperate woodlands of eastern and southern Asia (Hsu et al., 1994; Shi et al., 2006; Unver, 2007). Like other species of genus *Lycois, L. aurea* is very durable, tolerating the extremes of drought and waterlogging, as well as poor soil conditions (Wang et al., 2013; Xu et al., 2015). It also accumulates Amaryllidaceae alkaloids such as lycorine and galanthamine, which have been reported to exhibit medical values (Bores et al., 1996; Lilienfeld, 2002; Marco and do Carmo Carreiras, 2006; Lamoral-Theys et al., 2010). In general, Amaryllidaceae alkaloids are regarded as derivatives of the common precursor 4′-*O*-methylnorbelladine (Eichhorn et al., 1998; Bastida et al., 2011). There are three different groups of Amaryllidaceae alkaloids that are biosynthesized by three modes of intramolecular oxidative C–C phenol coupling (*para-ortho', para-para'* and *ortho-para'*) (Bastida et al., 2011). Although many detailed insights in biosynthetic steps of Amaryllidaceae alkaloids production have been revealed by the biochemical approaches labeling intermediates, the key enzymes involved in the biosynthesis of Amaryllidaceae alkaloids (especially for galanthamine) are far from being fully understood (Wang et al., 2013). In order to better identify the determinants in production of Amaryllidaceae alkaloids, studies on their inducible phenotype have been performed. For example, the improved production of Amaryllidaceae alkaloids was observed in some Amaryllidaceae plants when treated with elicitors including methyl jasmonate (MeJA), sodium nitroprusside (SNP) and ethylene (Colque et al., 2004; Mu et al., 2009; Ptak et al., 2010; Jiang et al., 2011). Known as a derivative of jasmonate metabolism, MeJA was shown to be an important signal molecules detected in a wide spectrum of plant species and function on a lot of biological processes including growth inhibition, senescence, wound response, plant defense and secondary mechanism (Wasternack, 2007; De Geyter et al., 2012; Wasternack and Hause, 2013). For example, three major classes of plant secondary metabolites defined as the terpenoids, alkaloids and phenylpropanoids were induced by jasmonates (De Geyter et al., 2012; Wasternack and Hause, 2013). Nevertheless, the detailed processes of MeJA-stimulated Amaryllidaceae alkaloids production and concomitant transcriptome changes associated with response to MeJA in *L. aurea* remain poorly understood. Furthermore, the precise regulation mechanisms controlling the biosynthesis of Amaryllidaceae alkaloids highly interconnected at the metabolic level and a possible transcriptional/post-transcriptional regulation still need to be elucidated.

RNA sequencing (RNA-seq) has been applied prevalently on transcriptomes analysis of various species for a wide range of purposes (Wang et al., 2009; Metzker, 2010; Stone and Storchova, 2015). The main outcome of RNA-seq data is to identify the differentially expressed genes, while it was also used to search for reference genes (Zhuang et al., 2015). Meanwhile, quantitative real-time polymerase chain reaction (qRT-PCR) technique, with quantitative accuracy, high sensitivity, low cost, and high-throughput characteristics, has also been widely used to determine gene expression levels and to validate transcriptomic data (Bustin, 2002; Radonic et al., 2004; Caldana et al., 2007; Van Guilder et al., 2008). For accurate qRT-PCR evaluation, it is necessary to select suitable reference genes as internal control under different experimental conditions because several factors including the starting material, RNA integrity, reverse transcription efficiency, cDNA quality, sample amount, and/or extraneous tissue and cell activities can significantly influence the accuracy of gene expression (Bustin, 2002; Huggett et al., 2005). In addition, gene expression can be highly tissue-specific and often varies based on the physiological status of the organism or experimental treatments, and not a single gene can act as a universal reference reported so far (Nicot et al., 2005; Gutierrez et al., 2008a; Gimeno et al., 2014; Zhuang et al., 2015). Hence, the selection of appropriate reference genes is important for obtaining valid results and proper interpretation from the analysis (Bustin, 2002; Bustin et al., 2009). Numerous studies have documented the selection of reference genes in various plants including *Arabidopsis* (Czechowski et al., 2005; Remans et al., 2008; Hong et al., 2010; Lilly et al., 2011), grasses (Hong et al., 2008; Lee et al., 2010), fruits (Reid et al., 2006; Tong et al., 2009; Clancy et al., 2013; Die and Rowland, 2013; Imai et al., 2014), vegetables (Expósito-Rodríguez et al., 2008; Wan et al., 2010; Xu et al., 2012), some desert plants (Li et al., 2012, 2015; Zhu et al., 2013), and crops such as soybean (Jian et al., 2008; Libault et al., 2008; Hu et al., 2009; Kulcheski et al., 2010), rice (Kim et al., 2003; Jain et al., 2006; Narsai et al., 2010), wheat (Paolacci et al., 2009), barley (Burton et al., 2004), buckwheat (Demidenko et al., 2011), potato (Nicot et al., 2005), and sugarcane (Iskandar et al., 2004). Of some *Lycoris* species, comparison and selection of reference genes in different tissues and floral development stages has been performed (Cui et al., 2011; Jiang et al., 2015). However, none has been assessed for a systematic selection of reference genes in *L. aurea* under abnormal condition (especially for abiotic stress and hormone treatments). Beside, one of our major research interests concerns the study of the galanthamine biosynthesis pathways, and we have characterized a number of candidate genes referring to this process in *L. aurea* (Wang et al., 2013). The need for suitable reference genes is thus urgent for qRT-PCR detection of gene expression especially regarding galanthamine biosynthesis and its regulation processes under various experimental conditions in *L. aurea*.

In this study, we selected 14 candidate reference genes based on the transcriptome datasets of *L. aurea* by RNA-seq (Wang et al., 2013; unpublished data). The expression profiles of these candidate reference genes were tested under various treatments and further validated the expression stability by qRT-PCR and evaluated using statistical algorithms including geNorm (Vandesompele et al., 2002), NormFinder (Andersen et al., 2004), and BestKeeper (Pfaffl et al., 2004). The comprehensive stability ranking of these reference genes under each specific experimental condition were also performed. Additionally, one target gene, secologanin synthase (cytochrome P450 *CYP72A1*), was used to validate the effectiveness of the selected reference genes. Finally, this work provides the basis for further research in exploring gene expression profiling and the regulation mechanism of galanthamine biosynthesis of *L. aurea* under different experimental conditions.

## Material and methods

### Plant materials and stress treatments

The bulbs of *Lycoris aurea* with the same or similar sizes (1.8–2.2 cm) in diameter were planted and grown in a greenhouse at the research station of Institute of Botany, Jiangsu Province and Chinese Academy of Sciences (118°83′ E; 32°05′ N), Nanjing, China. For various abiotic stress and hormone treatments, seedlings (with 2–3 leaves) were transferred into plastic pot (15.0 cm top diameter and 14.5 cm deep) containing half-strength Hoagland's nutrient solution. Plants were placed in a chamber with a mean temperature of 25.0 ± 1.4°C, a relative humidity of 60.0% ± 10%, and a day/night rhythm of 14/10 h. After 7 days maintenance, *L. aurea* seedlings were subjected to various experimental treatments following the methods described previously (Tian et al., 2015; Xiao et al., 2015). For drought treatment, 20% PEG-6000 solution (w/v, polyethylene glycol, Sangon, China) was applied to incubate the plants for 0, 1, 6, and 24 h. For cold and heat stress, Plants in the pots were placed at chamber with the temperature of 4°C and 42°C respectively for 0, 1, 6, and 24 h. For salinity treatment, seedlings were transferred to solution with 400 mM NaCl for 0, 1, 6, and 24 h. For sodium nitroprusside (SNP) and hormone treatments, plants were imposed in 500 μM SNP, 100 μM methyl jasmonate (MeJA) or 100 μM abscisic acid (ABA) for 0, 1, 6, and 24 h. MeJA (containing 0.02% (v/v) absolute ethanol and 0.02% (v/v) Tween-20), SNP and ABA were dissolved in distilled water. After that, the roots were sampled separately at different periods for each treatment used for expression analysis. *L. aurea* is a groundcover plant and typical hysteranthous geophyte appearing in autumn. Its floral stems and flowers start growing from August to September, and the leaves grow from September to October. So for tissue samples subset, six parts: filament, anther, column, petal, ovary and stem were collected when *L. aurea* is flowering. After the flowers wilted, root, bulb and leaf from the same plant were collected respectively. The detailed information of samples collected from various tissues/experimental conditions were also listed in Table S1. All samples were harvested from three replicate plants, frozen in liquid nitrogen and then stored at −80 °C prior to RNA isolation.

### Total RNA isolation and cDNA synthesis

The total RNA was extracted from the samples using RNAprep Pure Plant Kit (Tiangen Biotech, Beijing, China). To eliminate DNA contamination, total RNA was digested with DNase I (Ambion, USA) and then purified according to the manufacturer's protocol. The integrity of total RNA samples was verified by performing 1.5% (w/v) agarose gel electrophoresis, and the quantity and quality of RNA samples were measured with the NanoDrop 2000 Spectrophotometer (NanoDrop Technologies, ThermoScientific, USA). Only the RNA samples with absorption ratios of A260/280 = 1.8−2.2 and A260/230 higher than 1.8 were used for cDNA synthesis.

In order to perform qRT-PCR, an aliquot of 1 μg of total RNA was used for cDNA synthesis with a final volume of 20 μL using PrimeScript™ RT reagent Kit with gDNA Eraser (TaKaRa Bio Inc., Dalian, China) following the manufacturer's instructions. The cDNA was diluted 10-fold with nuclease-free water for qRT-PCR.

### Selection of candidate reference genes and primer design

We performed transcriptome sequencing of *L. aurea* root samples exposed to 100 μM MeJA at 0 (control) and 6 h (MJ100) using Illumina paired-end sequencing technology on an Illumina Hi-Seq™ 2000 platform. After assembly and annotation, the read counts of unigenes were converted to fragments per kilobase of exon model per millon mapped reads (FPKM values). To estimate expression stability of each gene, the following indices of FPKM values, including mean expression value (MV), standard deviation (SD) and coefficient of variation (CV) value (dividing SD by MV) were calculated according to the methods described previously (de Jonge et al., 2007; Zhuang et al., 2015). Based on previous qRT-PCR reports in the model plants *Arabidopsis*, 14 corresponding unigenes, which have credible protein annotation (Nr and Swiss-Prot databases), appropriate expression level, and a low CV of FPKM, were screened and selected from the *L. aurea* transcriptome for candidate reference genes (Table S2). According to the sequences of these unigenes (Data Sheet 1), specific primers were designed using Primer-BLAST in NCBI. The criteria for primer design were set as follows: primer lengths of 20–24 bp, GC contents of 45–55%, melting temperature (Tm) in a range of 55−60°C and amplicon lengths of 100–250 bp.

### Quantitative real-time PCR analysis

qRT-PCR was conducted in 96-well plates and performed on the LightCycler 480 (Roche Molecular Biochemicals, Mannheim, Germany). The reaction mix contained 2 μL diluted cDNA, 7.5 μL AceQ qPCR SYBR Green Master Mix (Vazyme, Nanjing, China), 0.4 μM of each primer and ddH_2_O in a final volume of 15 μL. Two biological replicates for all of the samples and three technical replicates of each biological replicate with a no-template control (NTC) were used. The qRT-PCR protocol was as follows: 95°C for 10 min, 40 cycles of 95°C for 15 s, 60°C for 30 s. To verify the specificity of each primer, a melting-curve analysis was included. The mean amplification efficiency of each primer pair was checked by the LinRegPCR program (Ruijter et al., 2009).

### Ranking the stabilities of candidate reference genes

Three software programs, geNorm (Vandesompele et al., 2002), NormFinder (Andersen et al., 2004) and BestKeeper (Pfaffl et al., 2004) were used statistically to rank the stability of the 14 selected reference genes across all the experimental subsets. All analyses using these packages occurred according to the manufacturers' instructions. For geNorm and NormFinder algorithms, the raw cycle threshold (Ct) value of each gene were transformed into relative expression levels according to the formula 2^−ΔCt^ (ΔCt = Ct value of each sample - the minimum Ct value). Then, the relative expression values were imported into geNorm and NormFinder to analyze gene expression stability. According to the geNorm manual, the average expression stability value (M) and pairwise variation (Vn/n+1) value for each reference gene with all other genes were automatically analyzed and ranked according to their expression stability. As recommended by geNorm, the threshold of *M* value was set as 1.5; a lower value of M indicated higher gene's expression stability. The Vn/Vn+1 value determines the optimal number of reference genes for accurate normalization. A cut-off value of Vn/n+1 < 0.15 indicates that an additional reference gene makes no significant contribution to the normalization (Vandesompele et al., 2002). The NormFinder program calculates a stability value (SV) for evaluating expression variation when using reference genes for normalization and the lower SV indicates the higher stability (Andersen et al., 2004). The BestKeeper is an Excel-based tool that is similar to geNorm and NormFinder, and it determines the stability ranking of the reference genes based on the coefficient of variance (CV) and the standard deviation (SD) of the average Ct values. The most stable gene exhibits the lowest CV ± SD value, and genes with SD greater than 1 were considered unacceptable and should be excluded (Pfaffl et al., 2004; Chang et al., 2012; Xiao et al., 2015). Three results of the stability rankings were integrated, generating a comprehensive ranking according to the geometric mean of three algorithms corresponding rankings.

### Validation of reference genes

To validate the reliability of the qRT-PCR data, we analyzed the expression profiles of the 14 candidate reference genes in RNA-seq and ranked them according to the CV of FPKM. The gene with the lowest CV was regarded as the most stable gene, which was double-checked by RNA-seq and qRT-PCR data in the MeJA treatment experiment. Meanwhile, the expression pattern of a target gene, secologanin synthase (*CYP72A1*) were analyzed using the most stable and least stable reference genes after normalization across all the experimental sets. For MeJA treatment subset, the expression levels of the target gene *CYP72A1* in qRT-PCR were also compared with the FPKM values in RNA-seq data. The sequences of these reference genes were obtained from the transcriptome data (**Text 1 in** Data Sheet 1). The amplification efficiencies of the target genes were also estimated by the LinRegPCR program. The average Ct value was calculated from three biological and technical replicates and used for relative expression analyses. The relative expression data were calculated according to the 2^−ΔCt^ method and presented as fold change (Livak and Schmittgen, 2001).

## Results

### Selection of candidate reference genes in *Lycoris aurea*, evaluation of amplification specificity and PCR efficiency

Fourteen candidate reference genes were chosen according to described *Arabidopsis* most stable genes and reference genes commonly used in qRT-PCR studies (Czechowski et al., 2005). Orthologous *L. aurea* sequences were retrieved after performing BLASTX on *L. aurea* transctiptome datasets (Wang et al., 2013; unpublished data). The qRT-PCR primer sequences and amplicon characteristics of 14 candidate reference genes are described in Table 1. The expression stability of candidate reference genes (*GAPDH, ACT*, α*-TUB*, β*-TUB, EF1-*α, *UBC, EXP1, EXP2, CYP1, CYP2, TIP41, PTBP1, YLS8*, and *PP2A*) named according Arabidopsis orthgologs and Nr annotation, was assessed under various conditions, such as abiotic stress (NaCl, PEG, heat, and cold stress), hormone treatment, and different tissues. The primer specificities were confirmed by agarose gel electrophoresis, sequencing and melting curves, which showed the single amplicon of the expected size and the single peak melting curves (Figure S1; Data sheet 1). The qRT-PCR products ranged from 137 to 213 bp. For each gene, the mean PCR efficiency of each primer pair ranged from 1.775 to 1.880, and the linear R^2^ (correlation coefficients) ranged from 0.9989 to 0.9997 (Table 1).

**Table 1:** **Genes and primer sets used for qRT-PCR**.

**Gene name**	**Gene ID**	**Gene symbol**	***Arabidopsis* homolog locus**	**Primer sequence (forward/reverse)**	**Size (bp)**	**E (%)**	***R*^2^**
Glyceraldehyde-3-phosphate dehydrogenase	CL7040.Contig2_All	*GAPDH*	*AT1G13440*	AAATTAAGGCTGCAATCAAG CAAGCCACAAGCTTCACAAAGT	169	1.874	0.9992
Actin	CL1144.Contig2_All	*ACT*	*AT3G18780*	CAAATCATGTTCGAGACCTTCA AGACGAAGAATGGCATGGGGA	173	1.873	0.9989
Ubiquitin-conjugating enzyme	CL5627.Contig8_All	*UBC*	*AT4G27960*	TGCCTTGACCATCTCCAAGGTT CTCAACTATCCATCCGCTCACCC	200	1.865	0.9995
Elongation factor 1-α	CL3597.Contig3_All	*EF1-α*	*AT1G07920*	AAGGATGGGCAGACCCGTGAGCA CCAACCTTCTTGAGGTAGGAAG	161	1.823	0.9991
α-tubulin	CL544.Contig2_All	*α-TUB*	*AT1G50010*	TGTGCATTGGTATGTTGGTGA GTCATCCCCCTCGTCACCCTC	139	1.872	0.9994
β-tubulin	Unigene13345_All	*β-TUB*	*At5g12250*	TATCAACAGTATCAGGATGCGAC CGAACAATCAAAACCACCATAA	213	1.853	0.9995
Serine/threonine-protein phosphatase PP2A	CL6570.Contig3_All	*PP2A*	*AT1G13320*	GTACCGAACATTAAATTCAAT CTTGATTTGCAAAATATCTGAC	176	1.863	0.9994
Expressed protein 1	CL7794.Contig1_All	*EXP1*	*AT2G32170*	ATTGAAACAACCTACACCGCAA GCTGTAAGAATGCTAATCGTTCA	133	1.872	0.9995
Polypyrimidine tract-binding protein 1	CL6418.Contig1_All	*PTBP1*	*AT3G01150*	GCAATTTTTGAGAAGAATGGTG GACAGATGAAGCTTACAGTAAC	131	1.876	0.9996
Expressed protein 2	CL670.Contig8_All	*EXP2*	*AT4G33380*	AAACATCAAGAGTGCTGGC TTGCATGCATAGAGTGATTAC	198	1.779	0.9996
TIP41-like protein	Unigene3100_All	*TIP41*	*AT4G34270*	GCAACCATCCAAAGTTTAACTGCT AATGTGCAAGCAGGGCTAGTAA	157	1.847	0.9996
Cyclophilin 1	CL6321.Contig1_All	*CYP1*	*At2g16600*	TCGTGAGGGCCATCGAGAAGGT CTCATAACAAACAGACCATTATT	137	1.819	0.9997
Cyclophilin 2	CL8032.Contig1_All	*CYP2*	*At4g33060*	TCCCGATTCTTCTGGAAAGG AAGCCTTGTCTGGTAGAACAGC	181	1.880	0.9996
mitosis protein *YLS8*	CL7523.Contig2_All	*YLS8*	*AT5G08290*	CGACTGGGACGAAACCTGCATGC GGATCGTACGCTCGTACATTG	138	1.775	0.9996
secologanin synthase	CL4267.Contig5_All	*CYP72A1*	*AT3G14690*	TTCACTCTCCCTTCTCCTTTA GCACCGATTTCCTCTTTCAT	150	1.820	0.9996

*Unigenes were selected from the transcriptome of L. aurea and searched in sequences of Arabidopsis thaliana using Blastn in GenBank. The sequences of Unigenes can be found in Data Sheet 1. The mean efficiency (E) of PCR amplification and the regression coefficient (R2) for each primer pair were calculated by LinRegPCR software*.

### Expression profiles of the candidate reference genes

**Figure 1:**
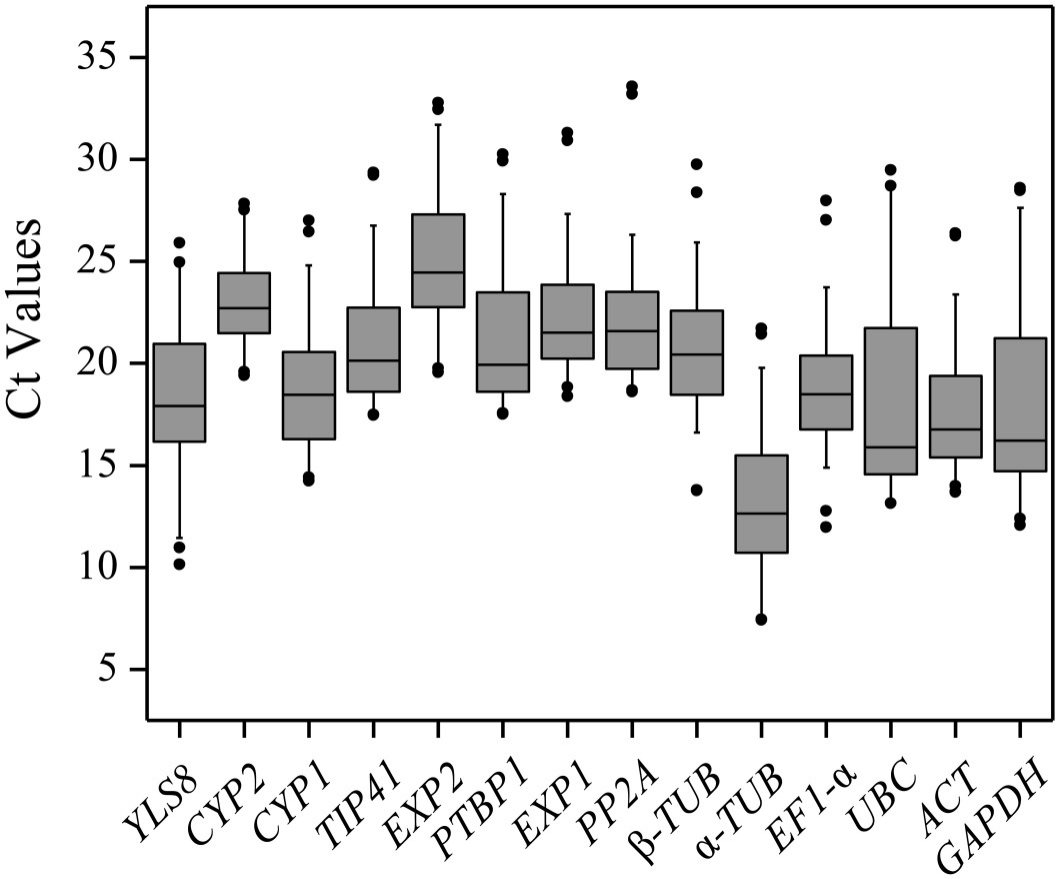
**Distribution of Ct values of candidate reference genes across all of the samples**. The Box-plot contains the mean, interquartile range, non-outlier range, and outlier.

To evaluate stability of the reference genes across all experimental samples, the transcript abundances of the 14 candidate reference genes were detected by their mean Ct values. The mean Ct values for the 14 candidate reference genes ranged between 13 and 25, with most lying between 18 and 22 across all tested samples. The average Ct value of all the tested samples was 22.9 cycles. Across all samples, α*-TUB* was the most abundantly expressed gene, with the lowest average Ct values of 13.44 ± 3.29 (mean ± SD), followed by *UBC* (17.80 ± 4.37), *ACT* (18.02 ± 3.34), *GAPDH* (18.05 ± 4.70), *YLS8* (18.49 ± 3.31), *EF1-*α (18.79 ± 2.90), *CYP1* (18.91 ± 3.17), and β-*TUB* (20.79 ± 2.85). *EXP2* was found to have the lowest level of expression of any of the genes tested, with a mean Ct values of 25.01 ± 2.99, followed by *CYP2* (22.93 ± 1.88) and *PP2A* (22.39 ± 3.45) (Figure 1). The genes with higher SD of Ct values indicated more variable expression compared to these with lower SD. *CYP2* showed the smallest variation in gene expression (22.93 ± 1.88), while *GAPDH* with the most variable levels of expression (Figure 1).

### Expression stability analysis of the candidate reference genes

In order to obtain a reliable dataset of the optimal reference genes under our experimental conditions, three of the most popular software programs were used: geNorm, NormFinder, and BestKeeper.

#### geNorm analysis

By using geNorm to assess the best reference genes in *Lycoris aurea*, the Ct values were transformed to relative expression levels and then calculated according to the manual. The *M* value was calculated at each step during stepwise exclusion of the least stable reference gene until two best genes were obtained (Table S3). In our analysis, a chart of the *M* value was generated that indicated the stability rank of the tested genes (Figure S2). The top two reference genes for qRT-PCR normalization were *CYP1* and *TIP41* for NaCl stress, *EXP1* and *PTBP1* for PEG stress, *CYP2* and *UBC* for cold stress, *CYP1* and *YLS8* for heat stress, *EXP1* and *PTBP1* in ABA treatment, *CYP2* and *TIP41* in MeJA treatment, *PP2A* and *PTBP1* in SNP treatment, and *EXP1* and *TIP41* in different tissues (Figure S2). Additionally, in the context of the total sample set, *EXP1* and *TIP41* ranked as the most stable two genes. Therefore, these two reference genes were deemed the most suitable for the widest range of test conditions in the current study.

**Figure 2:**
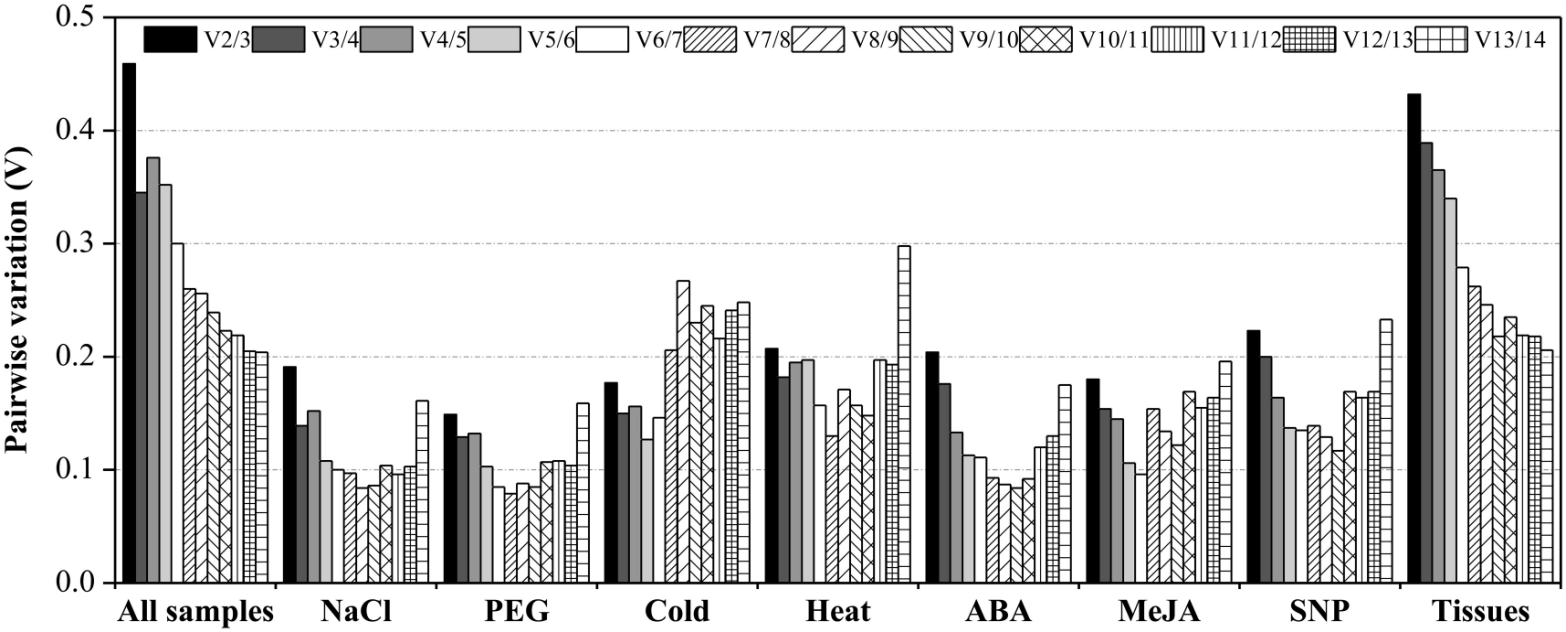
**Pairwise variation (Vn/Vn+1) values in nine subsets calculated by using geNorm**. The cut-off value to determine the optimal number of reference genes for qRT-PCR normalization is 0.15.

The Vn/n+1 between normalization factors calculated by the geNorm algorithm also determines the optimal number of reference genes for accurate normalization. A cut-off value of Vn/n+1 < 0.15 indicates that an additional reference gene makes no significant contribution to the normalization (Vandesompele et al., 2002). In the subset of PEG stress, the V2/3 value was below 0.15 (0.149), which suggested that two reference genes should be used for normalization. In the NaCl stress subset, three reference genes were sufficient for accurate normalization, as the V3/4 value was lower than 0.15. In the ABA and MeJA treatment subsets, four reference genes were needed for accurate normalization, as the V4/5 value was lower than 0.15. When the SNP-treated and cold-stressed samples were taken into account, the V5/6 value was lower than the cut-off value of 0.15, which indicated that five genes were suitable for all samples in this study. Additionally, when tissue and total samples were considered, the lowest pairwise variation value was still above 0.15 (Figure 2; Table S4).

#### NormFinder analysis

The results of the candidate reference gene stability ranking constructed by Normfinder are shown in Figure S3 for each subset and the SV is provided in Table S5. For all samples subset, NormFinder demonstrated that *EXP1* was the most stable (also ranked first by geNorm) followed by *TIP41, CYP1, PTBP1*. Similar to geNorm, *PP2A* was the least stable gene. In the subset of NaCl stress, *EXP1* and *UBC* were the most stable. In the PEG-stressed, cold-stressed and MeJA-treated subsets, *UBC* was the most stable gene. For ABA-treated subset, NormFinder suggested that *EXP1* and *PTBP1* were the most stable genes, and α*-TUB* was the least stable. In heat stress subset, *EXP1* and *PTBP1* were the most stable. *GAPDH* was the best gene for the SNP group. For the tissue samples subset, *EXP1* was the most stably expressed gene.

#### BestKeeper analysis

In both the NaCl stress subset and SNP treatment subset, *PTBP1* with the lowest CV ± SD values of 1.80 ± 0.36 and 3.19 ± 0.60, respectively, were identified as the most stable gene. In the PEG stress subset, *PTBP1* (2.93 ± 0.57) and *EXP1* (2.93 ± 0.60) were identified as the best reference genes for normalization. In the cold stress subset, *TIP41* (3.82 ± 0.75) had the lowest CV ± SD values, and showed remarkably stable expression. In the heat stress subset, only *ACT* showed SD < 1, which were considered as the most stable genes. In the ABA-treated subset, *ACT* had the lowest CV ± SD values of 2.70 ± 0.43, and showed the most stable expression. In MeJA-treated subset, the BestKeeper analysis suggested that *EXP1* was the most stable reference gene followed by *TIP41* and *CYP1*, while α*-TUB* was the least stable reference gene. Additionally, in both the all samples subset and tissue samples subset, no reference gene showed SD < 1, while *CYP2* had the lowest CV ± SD values of 6.62 ± 1.52 and 5.51 ± 1.34, respectively, were considered as the most stable genes (Table S6; Figure S4).

#### Comprehensive stability analysis of reference genes

**Figure 3:**
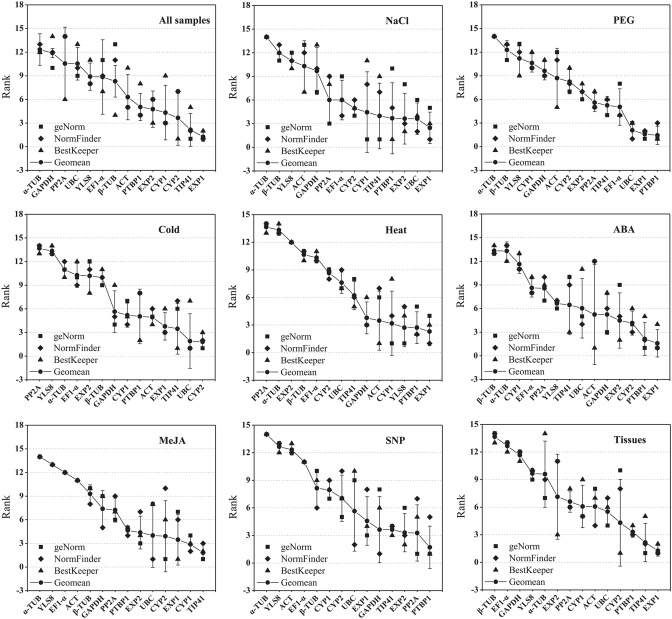
**Aggregation of three rankings**. The comprehensive ranking of candidate genes was calculated by the geometric mean of three types of rankings (geNorm, NormFinder, and BestKeeper) in each subset.

To obtain a consensus result of the most stable reference genes as recommended by the three methods according to the RefFinder approach (Xie et al., 2012; http://fulxie.0fees.us/?type=reference), the geometric mean of three algorithms corresponding rankings for each candidate gene were calculated (Table 2; Figure 3; Table S7). *EXP1* and *TIP41* were ranked as the top two stable reference genes in the all samples subset and tissue samples subset; *EXP1* also comprehensively ranked first in the NaCl stress subset, heat stress subset and ABA treatment subset. In cold stress subset, *CYP2* was stably expressed most. For both the heat stress subset and the SNP treatment subset, *PTBP1* was the most stable gene. Additionally, under MeJA treatment, *TIP41* was the best reference gene. The expression of *PP2A* was extremely unstable under cold stress and heat stress. α*-TUB* was unstably expressed in the majority of tested subsets, especially in the NaCl stress subset, PEG stress subset, MeJA treatment subset and SNP treatment subset.

**Table 2:** **Expression stability ranking of the 14 candidate reference genes**.

**Method**	**1**	**2**	**3**	**4**	**5**	**6**	**7**	**8**	**9**	**10**	**11**	**12**	**13**	**14**
**(A) RANKING ORDER UNDER ALL SAMPLES (BETTER-GOOD-AVERAGE)**														
geNorm	*EXP1/TIP41*		*CYP1*	*PTBP1*	*ACT*	*EXP2*	*CYP2*	*YLS8*	*UBC*	*GAPDH*	*EF1-α*	*α-TUB*	*β-TUB*	*PP2A*
NormFinder	*EXP1*	*TIP41*	*CYP1*	*PTBP1*	*ACT*	*EXP2*	*CYP2*	*YLS8*	*EF-1α*	*UBC*	*β-TUB*	*GAPDH*	*α-TUB*	*PP2A*
BestKeeper	*CYP2*	*EXP1*	*EXP2*	*β-TUB*	*TIP41*	*PP2A*	*EF1-α*	*PTBP1*	*CYP1*	*ACT*	*YLS8*	*α-TUB*	*UBC*	*GAPDH*
Comprehensive Ranking	*EXP1*	*TIP41*	*CYP2*	*CYP1*	*EXP2*	*PTBP1*	*ACT*	*β-TUB*	*EF1-α*	*YLS8*	*UBC*	*PP2A*	*GAPDH*	*α-TUB*
**(B) RANKING ORDER UNDER NACL STRESS (BETTER-GOOD-AVERAGE)**														
geNorm	*CYP1/ TIP41*		*PP2A*	*CYP2*	*EXP1*	*UBC*	*GAPDH*	*EXP2*	*EF1-α*	*PTBP1*	*β-TUB*	*YLS8*	*ACT*	*α-TUB*
NormFinder	*EXP1*	*UBC*	*EXP2*	*EF-1α*	*PTBP1*	*CYP2*	*TIP41*	*CYP1*	*PP2A*	*GAPDH*	*YLS8*	*ACT*	*β-TUB*	*α-TUB*
BestKeeper	*PTBP1*	*EXP2*	*EXP1*	*UBC*	*CYP2*	*EF1-α*	*ACT*	*PP2A*	*TIP41*	*YLS8*	*CYP1*	*β-TUB*	*GAPDH*	*α-TUB*
Comprehensive Ranking	*EXP1*	*UBC*	*EXP2*	*PTBP1*	*TIP41*	*CYP1*	*CYP2*	*EF1-α*	*PP2A*	*GAPDH*	*ACT*	*YLS8*	*β-TUB*	*α-TUB*
**(C) RANKING ORDER UNDER PEG STRESS (BETTER-GOOD-AVERAGE)**														
geNorm	*EXP1/PTBP1*		*UBC*	*TIP41*	*PP2A*	*EXP2*	*CYP2*	*EF1-α*	*GAPDH*	*CYP1*	*β-TUB*	*ACT*	*YLS8*	*α-TUB*
NormFinder	*UBC*	*EXP1*	*PTBP1*	*EF-1α*	*PP2A*	*TIP41*	*EXP2*	*CYP2*	*GAPDH*	*CYP1*	*ACT*	*YLS8*	*β-TUB*	*α-TUB*
BestKeeper	*PTBP1*	*EXP1*	*UBC*	*EF1-α*	*ACT*	*TIP41*	*PP2A*	*EXP2*	*YLS8*	*CYP2*	*GAPDH*	*CYP1*	*β-TUB*	*α-TUB*
Comprehensive Ranking	*PTBP1*	*EXP1*	*UBC*	*EF1-α*	*TIP41*	*PP2A*	*EXP2*	*CYP2*	*ACT*	*GAPDH*	*CYP1*	*YLS8*	*β-TUB*	*α-TUB*
**(D) RANKING ORDER UNDER COLD STRESS (BETTER-GOOD-AVERAGE)**														
geNorm	*CYP2/UBC*		*EXP1*	*GAPDH*	*ACT*	*TIP41*	*CYP1*	*PTBP1*	*β-TUB*	*EF1-α*	*α-TUB*	*EXP2*	*YLS8*	*PP2A*
NormFinder	*UBC*	*CYP2*	*EXP1*	*CYP1*	*GAPDH*	*ACT*	*TIP41*	*PTBP1*	*EF-1α*	*β-TUB*	*EXP2*	*α-TUB*	*YLS8*	*PP2A*
BestKeeper	*TIP41*	*PTBP1*	*CYP2*	*ACT*	*CYP1*	*EXP1*	*UBC*	*EXP2*	*GAPDH*	*α-TUB*	*β-TUB*	*EF1-α*	*PP2A*	*YLS8*
Comprehensive Ranking	*CYP2*	*UBC*	*TIP41*	*EXP1*	*ACT*	*PTBP1*	*CYP1*	*GAPDH*	*β-TUB*	*EXP2*	*EF1-α*	*α-TUB*	*YLS8*	*PP2A*
**(E) RANKING ORDER UNDER HEAT STRESS (BETTER-GOOD-AVERAGE)**														
geNorm	*CYP1/YLS8*		*GAPDH*	*EXP1*	*PTBP1*	*ACT*	*UBC*	*TIP41*	*CYP2*	*EF1-α*	*β-TUB*	*EXP2*	*α-TUB*	*PP2A*
NormFinder	*EXP1*	*PTBP1*	*GAPDH*	*CYP1*	*YLS8*	*TIP41*	*ACT*	*CYP2*	*UBC*	*EF-1α*	*β-TUB*	*EXP2*	*α-TUB*	*PP2A*
BestKeeper	*ACT*	*PTBP1*	*EXP1*	*YLS8*	*TIP41*	*GAPDH*	*UBC*	*CYP1*	*CYP2*	*β-TUB*	*EF1-α*	*EXP2*	*PP2A*	*α-TUB*
Comprehensive Ranking	*EXP1*	*PTBP1*	*YLS8*	*CYP1*	*ACT*	*GAPDH*	*TIP41*	*UBC*	*CYP2*	*EF1-α*	*β-TUB*	*EXP2*	*α-TUB*	*PP2A*
**(F) RANKING ORDER UNDER ABA TREATMENT (BETTER-GOOD-AVERAGE)**														
geNorm	*EXP1/PTBP1*		*GAPDH*	*CYP2*	*UBC*	*YLS8*	*PP2A*	*EF1-α*	*EXP2*	*TIP41*	*CYP1*	*ACT*	*β-TUB*	*α-TUB*
NormFinder	*EXP1*	*PTBP1*	*CYP2*	*UBC*	*EXP2*	*GAPDH*	*YLS8*	*EF-1α*	*TIP41*	*PP2A*	*CYP1*	*ACT*	*β-TUB*	*α-TUB*
BestKeeper	*ACT*	*EXP2*	*TIP41*	*EXP1*	*PTBP1*	*CYP2*	*YLS8*	*GAPDH*	*PP2A*	*EF1-α*	*UBC*	*α-TUB*	*CYP1*	*β-TUB*
Comprehensive Ranking	*EXP1*	*PTBP1*	*CYP2*	*EXP2*	*GAPDH*	*ACT*	*UBC*	*TIP41*	*YLS8*	*PP2A*	*EF1-α*	*CYP1*	*α-TUB*	*β-TUB*
**(G) RANKING ORDER UNDER MEJA TREATMENT (BETTER-GOOD-AVERAGE)**														
geNorm	*CYP2/TIP41*		*EXP2*	*CYP1*	*PTBP1*	*PP2A*	*EXP1*	*UBC*	*GAPDH*	*β-TUB*	*ACT*	*EF1-α*	*YLS8*	*α-TUB*
NormFinder	*UBC*	*CYP1*	*TIP41*	*PTBP1*	*GAPDH*	*EXP1*	*EXP2*	*β-TUB*	*PP2A*	*CYP2*	*ACT*	*EF-1α*	*YLS8*	*α-TUB*
BestKeeper	*EXP1*	*TIP41*	*CYP1*	*EXP2*	*PTBP1*	*CYP2*	*PP2A*	*UBC*	*GAPDH*	*β-TUB*	*ACT*	*EF1-α*	*YLS8*	*α-TUB*
Comprehensive Ranking	*TIP41*	*CYP1*	*EXP1*	*CYP2*	*UBC*	*EXP2*	*PTBP1*	*PP2A*	*GAPDH*	*β-TUB*	*ACT*	*EF1-α*	*YLS8*	*α-TUB*
**(H) RANKING ORDER UNDER SNP TREATMENT (BETTER-GOOD-AVERAGE)**														
geNorm	*PP2A/PTBP1*		*EXP1*	*TIP41*	*CYP2*	*EXP2*	*CYP1*	*GAPDH*	*UBC*	*β-TUB*	*EF1-α*	*ACT*	*YLS8*	*α-TUB*
NormFinder	*GAPDH*	*UBC*	*EXP2*	*TIP41*	*PTBP1*	*β-TUB*	*PP2A*	*EXP1*	*CYP1*	*CYP2*	*EF-1α*	*ACT*	*YLS8*	*α-TUB*
BestKeeper	*PTBP1*	*EXP2*	*TIP41*	*EXP1*	*PP2A*	*GAPDH*	*CYP2*	*CYP1*	*β-TUB*	*UBC*	*EF1-α*	*YLS8*	*ACT*	*α-TUB*
Comprehensive Ranking	*PTBP1*	*PP2A*	*EXP2*	*TIP41*	*GAPDH*	*EXP1*	*UBC*	*CYP2*	*CYP1*	*β-TUB*	*EF1-α*	*ACT*	*YLS8*	*α-TUB*
**(I) Ranking order UNDER DIFFERENT TISSUES (BETTER-GOOD-AVERAGE)**														
geNorm	*EXP1/TIP41*		*PTBP1*	*UBC*	*CYP1*	*PP2A*	*α-TUB*	*ACT*	*YLS8*	*CYP2*	*EXP2*	*GAPDH*	*EF1-α*	*β-TUB*
NormFinder	*EXP1*	*TIP41*	*PTBP1*	*ACT*	*CYP1*	*PP2A*	*UBC*	*CYP2*	*α-TUB*	*YLS8*	*EXP2*	*GAPDH*	*EF-1α*	*β-TUB*
BestKeeper	*CYP2*	*EXP1*	*EXP2*	*PTBP1*	*TIP41*	*UBC*	*ACT*	*PP2A*	*CYP1*	*YLS8*	*GAPDH*	*EF1-α*	*β-TUB*	*α-TUB*
Comprehensive Ranking	*EXP1*	*TIP41*	*PTBP1*	*CYP2*	*UBC*	*ACT*	*CYP1*	*PP2A*	*EXP2*	*α-TUB*	*YLS8*	*GAPDH*	*EF1-α*	*β-TUB*

### Reference genes validation

**Figure 4:**
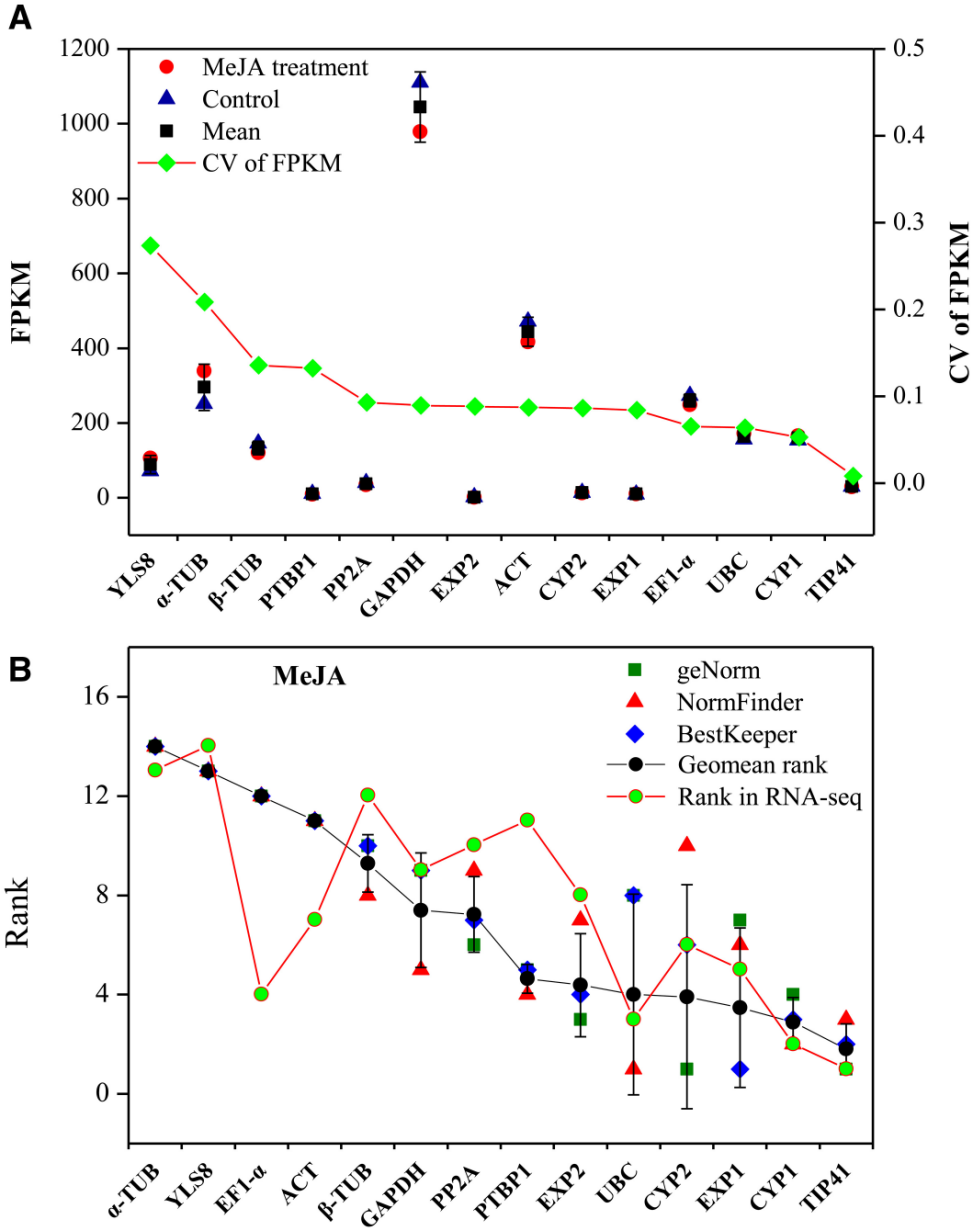
**Validation of qRT-PCR results through comparison with RNA-seq expression profiles. (A)** Stability ranking of candidate genes by CV of FPKM in RNA-seq. The gene with lower CV indicates more stable expression. **(B)** Correlation analysis between ranking of MeJA treatment subset by qRT-PCR and the ranking of RNA-seq. CV, coefficient of variation; FPKM, fragments per kilobase of exon model per million mapped reads.

To validate the selected reference genes, the stability of the candidate genes in qRT-PCR was compared with RNA-seq-based gene expression profiling. *L. aurea* samples treated with 100 μM MeJA and control were mapped, and their unigenes were quantified by RNA-seq as in this study (Table S2; unpublished data). FPKM represents the expression quantities of the unigenes, and the CV of FPKM represents the variability in gene expression. As shown in Figure 4A, *YLS8*, α*-TUB*, β*-TUB*, and *EXP2* showed a high CV value, indicating they were not stable genes. In contrast, *TIP41, PTBP1*, and *CYP1* had a lower CV, indicating that they were more stable under MeJA treatment. The ranking of gene stability in the MeJA treatment subset and the ranking of these genes in the RNA-seq data was also compared (Figure 4B). To some extent, the two types of rankings were consistent and had a positive correlation coefficient of *r* = 0.64. *CYP1 and TIP41*, which were two most stable genes in MeJA treatment were also shown relative stable expression through transcriptome analysis. Unstable genes, such as α*-TUB* and *YLS8* also had similar rank in MeJA treatment subset and transcriptome analysis.

**Figure 5:**
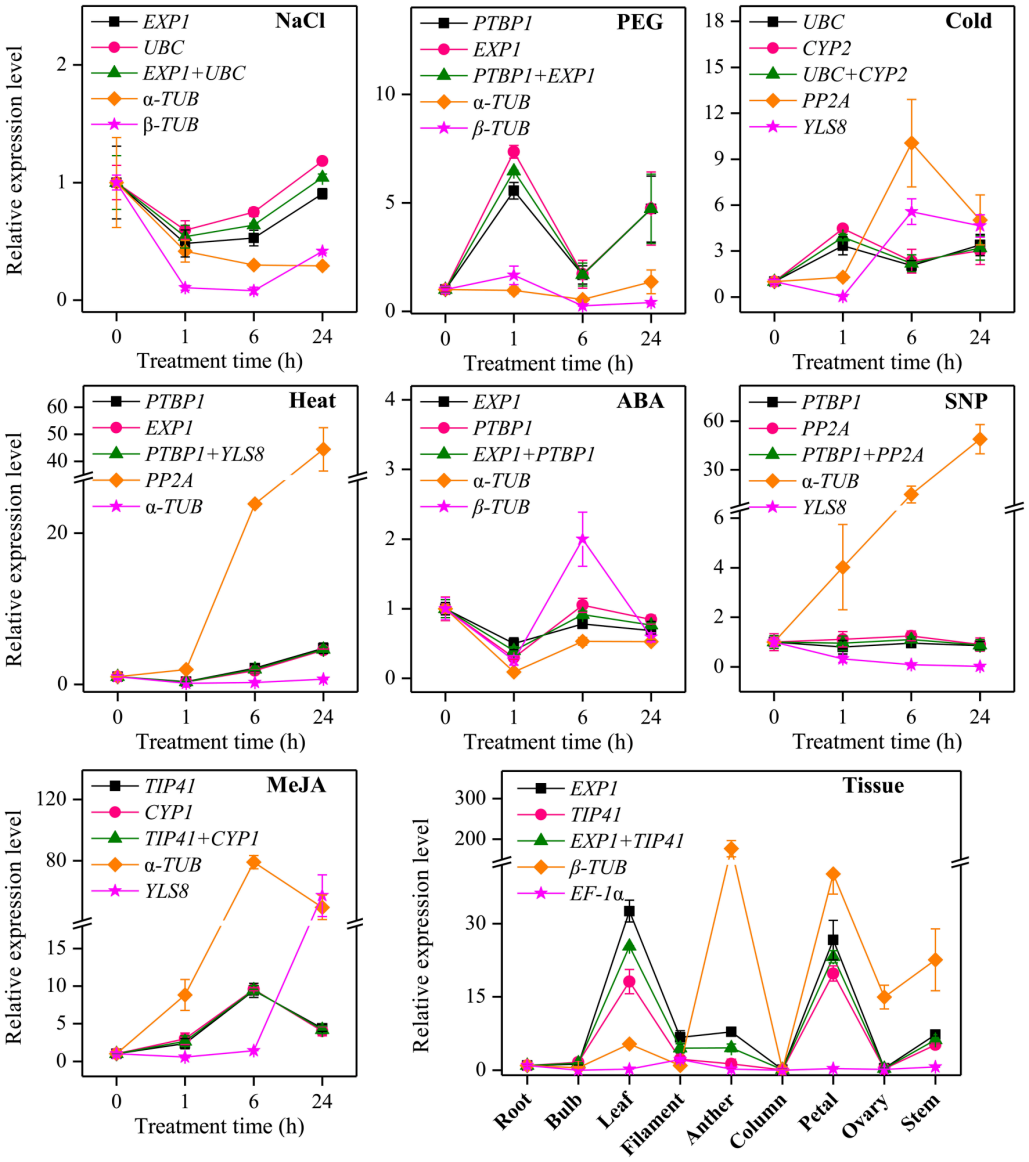
**Relative quantification of *CYP72A1* expression using the validated reference gene(s)**. The results are represented as mean fold changes in relative expression when compared to the first sampling stage (0 h). cDNA samples were taken from the same subset used for gene expression stability analysis. Roots were collected from *L. aurea* seedlings subjected to various treatments after 0, 1, 6, and 24 h. For tissue samples, different tissue types were collected from different development stage of *L. aurea*.

Additionally, to further validate the selected reference genes, the relative expression levels of a target gene, *CYP72A1* in *L. aurea* under different experimental conditions were evaluated using qRT-PCR. It was normalized using the most stable reference genes found in each subset both singly and in combination as well as two least stable reference genes as an internal control (Figure 5). A substantial divergence can occur in the relative transcript abundance of *CYP72A1* when normalized to different kinds of reference genes. Under NaCl treatment, the expression level of *CYP72A1* was down-regulated first then increased when normalized using the two stable genes (*EXP1* and *UBC*), while the expression level was down-regulated dramatically when normalized using the least stable combination (α*-TUB* and β*-TUB*). Under PEG stress, the highest expression level of *CYP72A1* appeared at 1 h and was 4.56- and 6.35-fold higher than control (0 h) by using the most stable reference genes (*PTBP1* and *EXP1*) as the internal control. In response to cold stress, *CYP72A1* exhibited a clear up-regulation to the highest level by 1 h of treatment and then declined thereafter when the two most stable references genes (*UBC* and *CYP2*) were used, while the expression level of *CYP72A1* fluctuated dramatically during cold stress when the least stable reference genes (*PP2A* and *YLS8*) were used for normalization. We also examined the reference genes in the ABA treatment and heat stress subsets. The results showed that both *PTBP1* and *EXP1* could serve as stable reference genes for normalization, while the relative expression folds of *CYP72A1* normalized by α*-TUB* were slightly decreased compared to the stable genes *PTBP1* and *EXP1* under ABA treatment. With SNP treatment, the expression level of *CYP72A1* was not affected significantly when normalized using the two stable genes (*PTBP1* and *PP2A*), while the expression level was overestimated and underestimated when normalized using the least stable reference genes α*-TUB* and *YLS8* respectively. In the same way, for the MeJA treatment subset, the expression level of *CYP72A1* increased to a peak at 6 h and then decreased when normalized using two most stable genes (*TIP41 and CYP1*) and an unstable gene α*-TUB*, but the expression value of *CYP72A1* was much higher when normalized by α*-TUB*. Additionally, the maximum expression level occurred at 24 h when *YLS8* was used as a reference. To validate the results, the expression levels of *CYP72A1* in qRT-PCR were compared with the FPKM values in RNA-seq data under MeJA treament. When *TIP41* and *CYP1* were used as the reference genes, the expression level of *CYP72A1* was generally identified with the expression profile in RNA-seq (Figure S5). Contrastingly, when the least stable reference gene α*-TUB* was used as normalization factor, the expression level of *CYP72A1* was significantly overestimated (Figure 5). Our tissue type analysis revealed that the transcript abundance of *CYP72A1* was the highest in the leaf, followed by the petal when normalized by the most stable genes (*EXP1* and *TIP41*).

## Discussion

Due to its high sensitivity, specificity, accuracy and reproducibility, qRT-PCR is commonly regarded as the most appropriated method for high-throughput analysis of gene expression profiling (Gutierrez et al., 2008b). However, random selection of reference genes, which may be influenced by different tissue types and experimental treatments, could cause the misinterpretation of results (Dheda et al., 2005). Therefore, validation of suitable reference genes for data normalization is mandatory to obtain reliable results for each experimental condition in different species (Schmittgen and Zakrajsek, 2000).

In this study, we had performed the large-scale transcriptome data of *L. aurea* (Wang et al., 2013; unpublished data) serving as the source of the reference gene selection. The expression levels and stability of 14 candidate reference genes were measured in *L. aurea* roots submitted to different conditions as well as in different tissues of *L. aurea* seedlings. By using three different software programs (geNorm, NormFinder, and BestKeeper), the 14 candidate reference genes exhibited various performance in their stability in *L. aurea*. Additionally, because of the different algorithms, the rankings generated by the three softwares were not completely identical (Table 2; Figure 3; Table S7). For example, in the cold stress subset, *UBC* was ranked first by geNorm and NormFinder, while it was ranked seventh by BestKeeper. In the NaCl stress subset and heat stress subset, the most stable gene in geNorm, such as *CYP1*, was ranked at a medium position or even a bottom position in NormFinder or BestKeeper. This apparent divergence is probably due to the discrepancies in the three statistical algorithms to calculate stability (Niu et al., 2015). geNorm identifies two reference genes with the highest degree of similarity in expression profile and the lowest intra-group variation (Vandesompele et al., 2002; Jian et al., 2008; Cruz et al., 2009). In contrast, NormFinder takes both the inter- and intra-group variations into account, and combines them into a stability value, and finally ranks the top genes with minimal inter- and intra-group variation (Andersen et al., 2004). As for BestKeeper, it determines the stability ranking of the reference genes according to their CV and SD values (Chang et al., 2012; Xiao et al., 2015). It has been recommended more than two algorithms should be used for reference gene stability evaluation (Jacob et al., 2013; Štajner et al., 2013; Xiao et al., 2015). Besides, a comprehensive tool RefFinder was developed to generate the final overall ranking of tested reference genes based on the geometric mean of the weights of every gene calculated by each program (Xie et al., 2012). A lower geometric mean of rankings indicates that the gene is more stable, and more narrow error bars indicate that the result is more reliable (Xiao et al., 2015). Therefore, by calculating the geometric mean of three rankings, a clear comprehensive ranking for each gene was generated (Table 2; Figure 3; Table S7). It showed that the comprehensively ranked first gene *EXP1* in all samples subset, NaCl stress subset, heat stress subset, ABA treatment subset, and tissue samples subset has lower geometric mean and narrower error bars because it all ranked top in geNorm, NormFinder, and BestKeeper. It is more reliable that the ranked first gene was the relative most stable gene in corresponding subset. Specifically, we also noticed that β-*TUB* and *GAPDH* displayed relatively low expression stability in the tissue samples subset, which is similar to the previous result in *Lycoris longituba* (Cui et al., 2011).

Traditionally, reference genes are usually cellular maintenance genes, which play housekeeping roles in basic cellular components and functions, such as tubulin (*TUB*), elongation factor 1-α (*EF1-*α), glyceraldehyde-3-phosphate dehydrogenase (*GAPDH*), ubiquitin (*UBQ*), 18S ribosomal RNA (*18S rRNA*), and actin (*ACT*). However, a growing number of studies indicated that the expression levels of most of these classic reference genes are somewhat can vary greatly and are unsuitable for gene normalization in many species under specific conditions (Thellin et al., 1999; Czechowski et al., 2005; Nicot et al., 2005; Jian et al., 2008; Die et al., 2010; Yang et al., 2010; Lilly et al., 2011; Li et al., 2012; Zhu et al., 2013). So housekeeping genes used as internal reference genes in each species should be taken with some caution under a given subset of experimental conditions (Gutierrez et al., 2008a; Ma et al., 2013; Lin et al., 2014). In this study, *ACT* ranked neither the top three most stable genes nor the least stable genes under tested experimental conditions. *EF1-*α and *GAPDH* displayed a less stable expression pattern. α*-TUB* and β*-TUB* were always the least stable reference genes under almost all the tested experimental conditions. Additionally, *UBC*, an ubiquitin-conjugating enzyme gene, was ranked first in both PEG stress subset and cold stress subset. It was also top gene in the all samples subset (Table 2; Figure 3). The strong stability of *UBC* in *L. aurea* was consistent with the results in *Corchorus olitorius* and *Platycladus orientalis* (Chang et al., 2012; Niu et al., 2015); However, it was not satisfactory for qRT-PCR normalization in different tissues of bamboo (Fan et al., 2013).

Some studies have identified several new reference genes that could be more stably expressed under specific conditions as compared with classic ones (Czechowski et al., 2005; Libault et al., 2008; Løvdal and Lillo, 2009; Zhu et al., 2013). In the present study, four new reference genes *YLS8, PP2A, EXP1*, and *TIP41* were tested. *EXP1* was highly stable within developing stages of rice anthers and pollens (Ji et al., 2014). We also observed that *EXP1* was ranked first in the tissue samples subset as well as in the all samples subset, NaCl stress subset, heat stress subset, and ABA treatment subset. Meanwhile, it is notable that *EXP1* ranked head of *EXP2* in our study (Figure 3). The *YLS8* gene is also reported to be a stable reference gene (Morgante et al., 2011; Han et al., 2013; Štajner et al., 2013) while it was expressed stably only in the heat stress subset. Similar to chicory (Delporte et al., 2015), our results showed that *TIP41* is the most suitable gene among all the reference genes we have tested within samples elicited with MeJA. For the study with different tissues of *L. aurea, TIP41* was also identified as a good reference gene which ranked the second. It was also somehow stable in the cold stress subset, NaCl stress subset and SNP treatment subset (Figure 3). Former studies showed that *TIP41* was expressed stably at different developmental stages of olive plants and in various tissues of bamboo (Fan et al., 2013; Resetic et al., 2013). Although it was recommended as a reference gene under abiotic stress in *Brassica juncea* and *Salicornia europaea* (Chandna et al., 2012; Xiao et al., 2015), *TIP41* was suitable for *Coffea arabica* under nitrogen starvation, salt stress or heat stress (de Carvalho et al., 2013).

RNA-seq has been applied prevalently on analyzing the transcriptomes of various species for a range of purposes (Wang et al., 2009; Li et al., 2015; Stone and Storchova, 2015). A large amount of transcript information as well as expression profiling of thousands of genes could be obtained by RNA-seq. It was also used to search for reference genes. Any gene with a minimal expression level variation in every analyzed sample is considered as candidate reference gene. To validate the results of qRT-PCR, we compared the result with RNA-seq data whose samples were also under the MeJA treatment. The two results (qRT-PCR and RNA-seq) supported each other, as they had a significant positive correlation coefficient (*r* = 0.64; Figure 4). Therefore, the results of this experiment are credible. Additionally, under chosen experimental conditions, good reference genes are stably expressed and should have a kind of expression level comparable to those of the target genes. In this study, a validation test using the most stable reference genes found in each subset both singly and combined as well as two least stable genes as an internal control in qRT-PCR analysis of target gene expression patterns, *CYP72A1*, was also performed (Figure 5). Our results showed that expression of *CYP72A1* was induced by NaCl, Cold, and PEG stress as well as MeJA treatment. Additionally, when α*-TUB* and β*-TUB* was validated as a reference gene for normalization the target gene *CYP72A1*, the expression pattern was obviously overestimated or underestimated (Figure 5). So the appropriate selection of reference genes is critically important for the normalization of target gene expression with qRT-PCR in *Lycoris aurea*.

## Conclusion

The selection of suitable reference genes is a prerequisite to quantifying gene expression by qRT-PCR. Here, we presented a systematic attempt to validate a set of candidate reference genes for the normalization of gene expression using qRT-PCR in *L. aurea* subjected to a wide range of experimental conditions as well as across different tissues. The expression stability of the 14 candidates was analyzed by the three commonly used programs (geNorm, NormFinder, and BestKeeper), and their results were further integrated into a comprehensive ranking based on the geometric mean. For the study of gene expression under NaCl stress, we recommend *UBC* and *EXP1* to normalize the qRT-PCR data. For gene expression study under cold stress, *UBC* and *CYP2* are the two most suitable reference genes. We also got the stable reference genes as *EXP1* and *PTBP1* for heat stress, PEG stress and ABA treatment, *PTBP1* and *PP2A* for SNP treatment, and *TIP41* and *CYP1* for MeJA treatment, respectively. For gene expression study in the various tissues, *EXP1, TIP41*, and *PTBP1* are recommended as the best reference genes for normalization. In addition, the two least stable reference genes α-*TUB* and β*-TUB* should be carefully used for normalization. The reliability of these results was enhanced by comparing part qRT-PCR result with RNA-seq data, and the selected reference genes can significantly reduce errors in genes quantification. Our results demonstrate that transcriptome sequencing data is a useful source for candidate reference genes screening and signify the importance of identification of specific reference genes for specific conditions in *L. aurea*. Furthermore, the reference genes selected in current study will be helpful for accurate normalization of qRT-PCR data and facilitate the future work on gene expression studies in *L. aurea*.

## Author contributions

RW and BX designed the research; RM, SX, YZ performed most of the experiments and data analysis; RM, SX, RW wrote the draft of the paper. YZ and BX participated in the preparation of the manuscript. All authors have read and approved the final manuscript.

### Conflict of interest statement

The authors declare that the research was conducted in the absence of any commercial or financial relationships that could be construed as a potential conflict of interest.

## Supplementary material

Figure S1 **Melting curves and agarose gel electrophoresis of PCR products. (A)** Melting curves of 14 candidate reference genes tested in this study. **(B)** Agarose gel (1.5%) electrophoresis showing amplification of a single PCR product of the expected size. M represents DNA marker having bands of 2000 bp, 1500 bp, 1000 bp, 750 bp, 500 bp, 250 bp, and 100 bp.

Figure S2 **Expression stability values (M) and ranking of the candidate reference genes as calculated by geNorm**. A lower *M*-value indicates more stable expression.

Figure S3 **Stability value and ranking of the candidate reference genes based on NormFinder**. A lower stability value (SV) indicates more stable expression.

Figure S4 **Expression stability and ranking of the candidate reference genes calculated by BestKeeper**. CV, coefficient of variation; SD, standard deviation. The gene with lower CV and SD shows more stable expression.

Figure S5 **The expression level of the target gene *CYP72A1* under control and MeJA treatment in RNA-seq**. The samples exposed to control and MeJA at 6 h were used for transcriptome sequencing. FPKM: fragments per kilobase of exon model per million mapped reads.

Table S1 **The detailed information of samples collected from various tissues/experimental conditions**.

Table S2 **Selected unigenes from the transcriptome data of *Lycoris aurea***. The unigenes with appropriate expression quantities and highly reliable annotation were selected to evaluate the stability of gene expression.

Table S3 **geNorm ranking of 14 candidate reference genes**. *M*-value: expression stability value. The stability of genes was ranked according to the *M*-value.

Table S4 **Pairwise variation (Vn/n+1) analysis of 14 candidate reference genes calculated by geNorm to determine the optimal number of reference genes for normalization**. The threshold value was proposed to be 0.15, below which the inclusion of an additional reference genes is not necessary.

Table S5 **NormFinder ranking of 14 candidate reference genes**. SV: stability value. The stability of genes was ranked according to the SV.

Table S6 **BestKeeper ranking of 14 candidate reference genes**. CV, coefficient of variation; SD, standard deviation. The stability of genes was ranked according to CV and SD.

Table S7 **Comprehensive stability ranking of 14 candidate reference genes based on the results of the three software programs**. The comprehensive ranking of each gene was calculated by the geometric mean of the rankings in geNorm, NormFinder, and BestKeeper.

Data Sheet 1 **Sequences of unigenes in the transcriptome of *L. aurea***. Specific primers for qRT-PCR were designed based on these sequences in Text S1. Amplification of PCR products were verified by sequencing.
