# Supplementary figures and images for: Selection and Validation of Appropriate Reference Genes for Quantitative Real-Time PCR Analysis of Gene Expression in Lycoris aurea
Source: Front Plant Sci. 2016 Apr 25;7:536. doi: 10.3389/fpls.2016.00536 (PMC4843812; doi:10.3389/fpls.2016.00536)

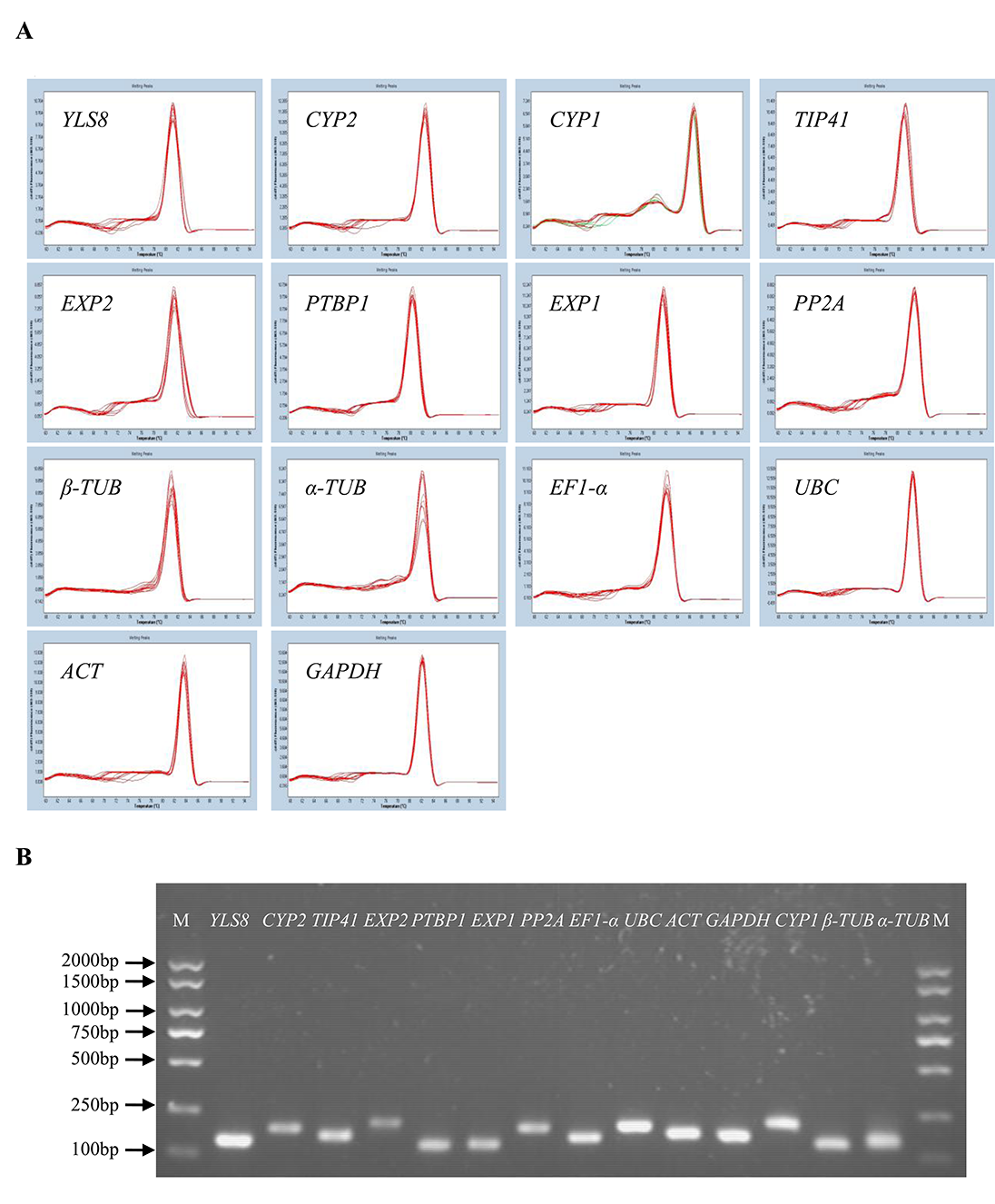

Supplement: Figure S1 — Melting curves and agarose gel electrophoresis of PCR products. (A) Melting curves of 14 candidate reference genes tested in this study. (B) Agarose gel (1.5%) electrophoresis showing amplification of a single PCR product of the expected size. M represents DNA marker having bands of 2000 bp, 1500 bp, 1000 bp, 750 bp, 500 bp, 250 bp, and 100 bp. [file Image1.TIFF]

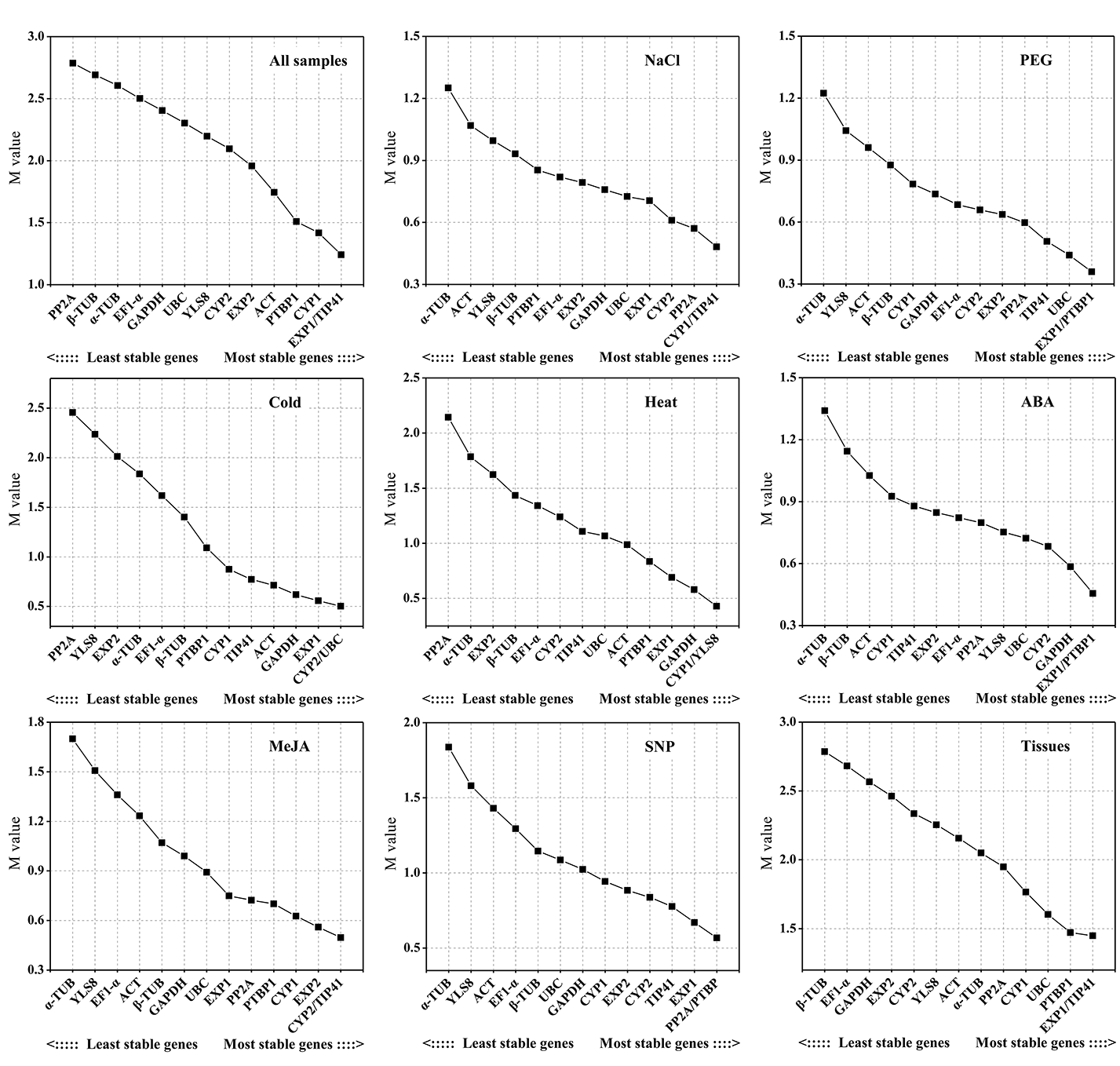

Supplement: Figure S2 — Expression stability values (M) and ranking of the candidate reference genes as calculated by geNorm. A lower M-value indicates more stable expression. [file Image2.TIFF]

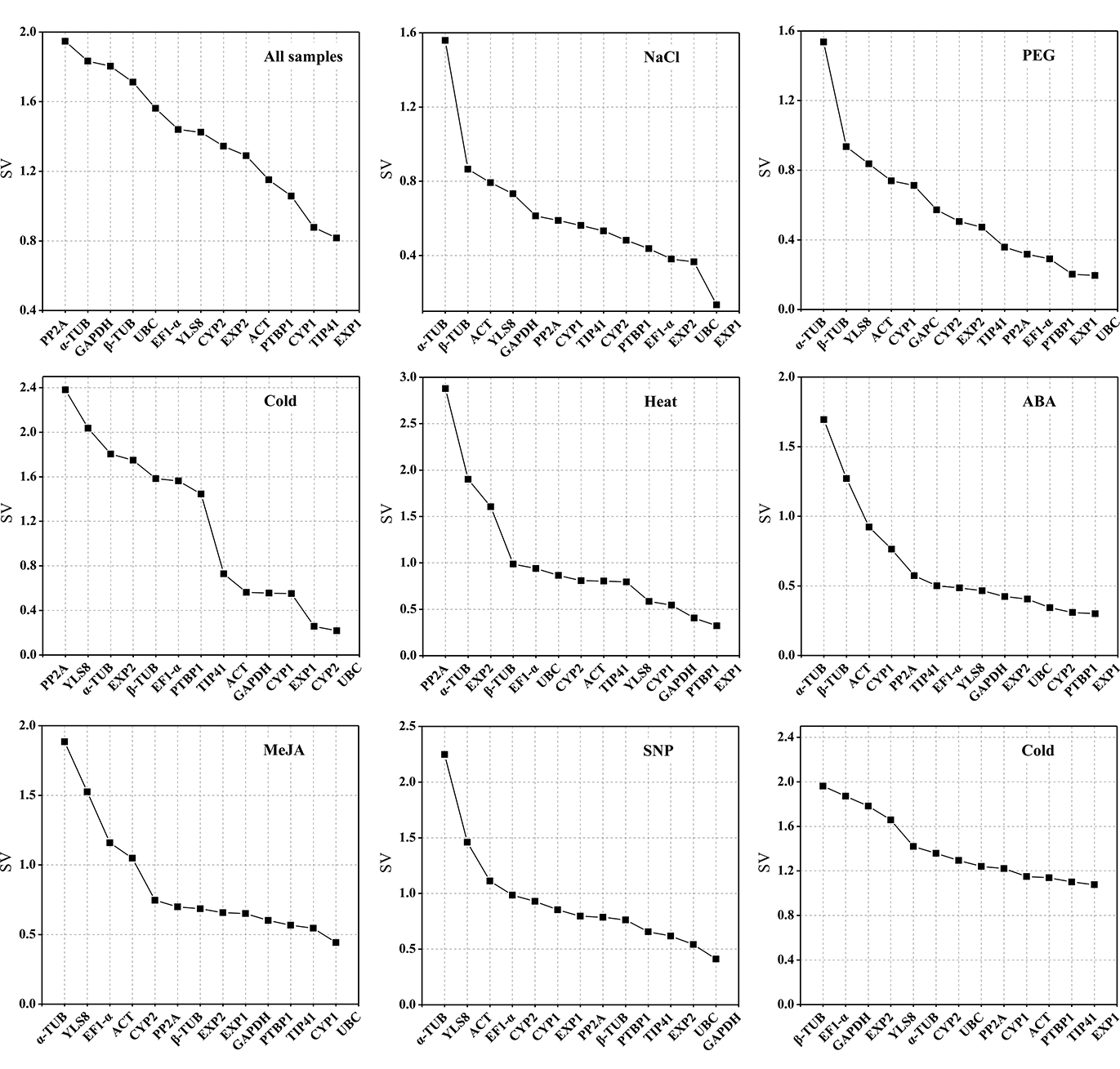

Supplement: Figure S3 — Stability value and ranking of the candidate reference genes based on NormFinder. A lower stability value (SV) indicates more stable expression. [file Image3.TIFF]

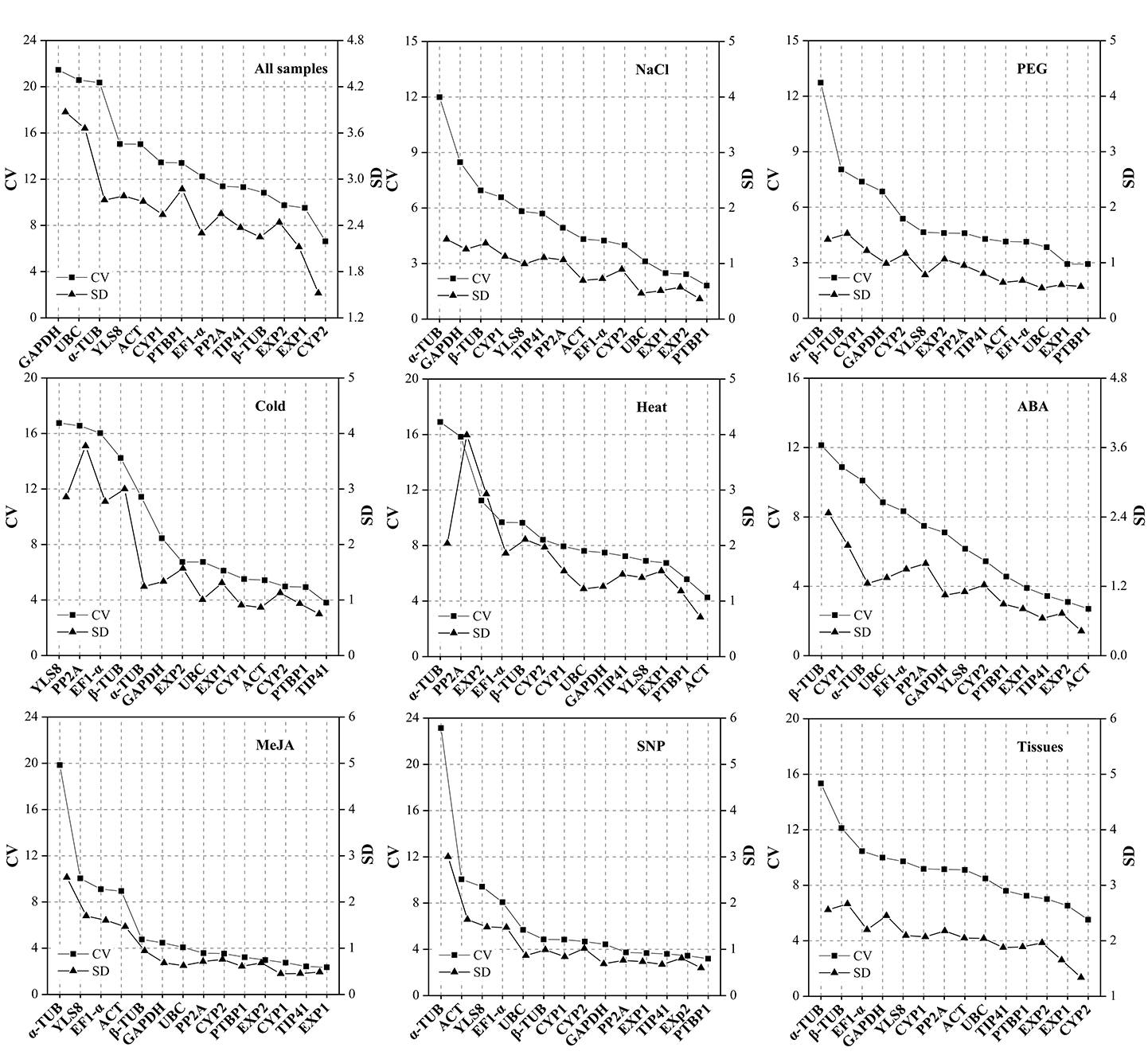

Supplement: Figure S4 — Expression stability and ranking of the candidate reference genes calculated by BestKeeper. CV, coefficient of variation; SD, standard deviation. The gene with lower CV and SD shows more stable expression. [file Image4.TIFF]

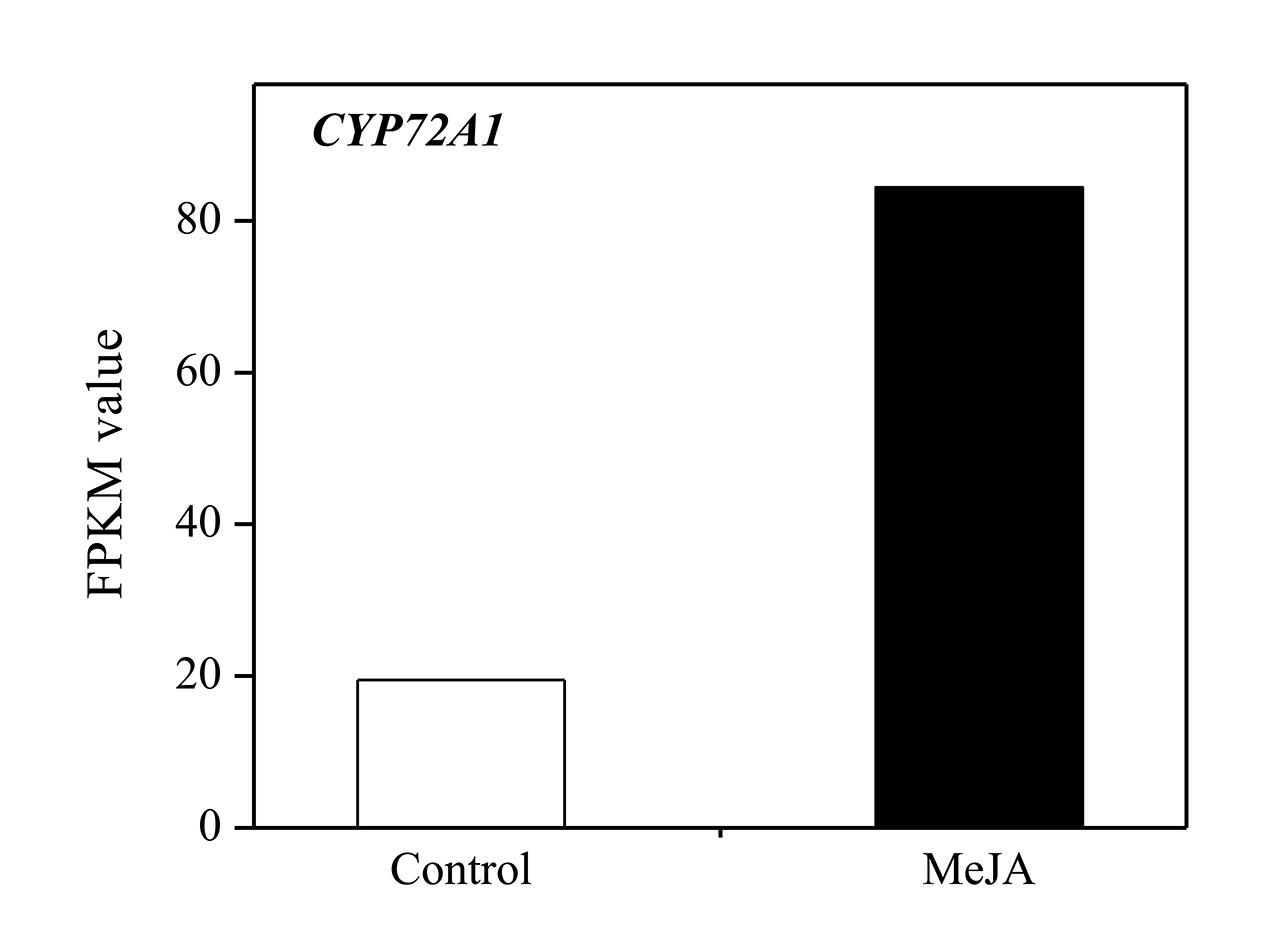

Supplement: Figure S5 — The expression level of the target gene CYP72A1 under control and MeJA treatment in RNA-seq. The samples exposed to control and MeJA at 6 h were used for transcriptome sequencing. FPKM: fragments per kilobase of exon model per million mapped reads. [file Image5.TIFF]
